# Supplementary material for: Patterns of Intron Gain and Loss in Fungi
Source: PLoS Biol. 2004 Nov 30;2(12):e422. doi: 10.1371/journal.pbio.0020422 (PMC532390; doi:10.1371/journal.pbio.0020422)
Supplement: Table S1 — Also available at http://genes.mit.edu/NielsenEtAl/. (4.3 MB ZIP). [file pbio.0020422.st001.zip › NielsenEtAl/html/993.html]

AN4182.1.NCU09778.1.MG01362.1.FG08468.1


```
 CLUSTAL W (1.82) Multiple Sequence Alignments - Introns Inserted


Sequence 1: NCU09778.1	328 aa
Sequence 2: MG01362.1	320 aa
Sequence 3: FG08468.1	325 aa
Sequence 4: AN4182.1	323 aa
Alignment Length: 331 aa
Number Identitical Residues: 235 aa
Alignment Score (without introns) 10081


MG01362.1 	MENYQKLEKIGEG1TYGVVYKARDLLNGGRIVAMKKIRLEAEDEGVPSTAIREISLLKEM
NCU09778.1	MENYQKLEKIGEG1TYGVVYKARDLANSGRIVALKKIRLEAEDEGVPSTAIREISLLKEM
FG08468.1 	MENYQKLEKIGEG1TYGVVYKARDLANGGRIVALKKIRLEAEDEGVPSTAIREISLLKEM
AN4182.1  	MENYQKIEKIGEG1TYGVVYKARELTHPNRIVALKKIRLEAEDEGVPSTAIREISLLKEM
          	******:****** *********:* : .****:**************************

MG01362.1 	RDPNIVRLFNIVHTDG---TKLYLVFEFLDLDLKKYMEALPVADGGRGKALPEGTGPQLS
NCU09778.1	RDPNIVRLLNIVHADGGQGHKLYLVFEFLDLDLKKYMEALPVSDGGRGKALPEGTGAGLH
FG08468.1 	RDPNIVRLFNIVHSDG---HKLYLVFEFLDLDLKKYMESLPISDGGRGKALPEGSSPHLQ
AN4182.1  	NDPNIVRLLNIVHADG---HKLYLVFEFLDLDLKKYMEALPVSEGGRGRALPDGS-TLSR
          	.*******:****:**    ******************:**:::****:***:*: .   

MG01362.1 	RLGLGDTMIKKFMSQLCDGVRYCHSHRILHRDLKPQNLLIDREGNLKLADFGLARAFGVP
NCU09778.1	NLGLGEDIIKKFMSQLCEGVRYCHSHRVLHRDLKPQNLLIDRDGNLKLADFGLARAFGVP
FG08468.1 	HLGLGDTVVRKFMYQLCDGVKYCHSHRVLHRDLKPQNLLIDKDGNLKLADFGLARAFGVP
AN4182.1  	NLGLGDAMVKKFMAQLIEGIRFCHSHRVLHRDLKPQNLLIDRDGNLKLADFGLARAFGVP
          	.****: :::*** ** :*:::*****:*************::*****************

MG01362.1 	LRTYTHE~VVTLWYRAPEILIGGRQYSTGVDMWSVGCIFAEMCTRKPLFPGDSEIDEIFK
NCU09778.1	LRTYTHE~VVTLWYRAPEILLGGRQYSTGVDMWSVGCIFAEMCTRKPLFPGDSEIDEIFK
FG08468.1 	LRTYTHE~VVTLWYRAPEILLGGRQYSTGVDMWSVGCIFAEMCTRKPLFPGDSEIDEIFK
AN4182.1  	LRTYTHE0VVTLWYRSPEILLGGRQYSTGVDMWSCGAIFAEMCTRKPLFPGDSEIDEIFK
          	******* *******:****:************* *.***********************

MG01362.1 	IFR2LLGTPTEETWPSVTDEHIYPDFKPSFPKWQRDPNMKLCPGLNDAGLDLLEMMLVYD
NCU09778.1	IFR2LLGTPTEDIWPGVTS---YPDFKASFPKWVRDYNVPLCQNLDDVGLELLESMLVYD
FG08468.1 	IFR2TLGTPSEDNWPGVTS---YPDFKASFPKWQRDYSKSLCSTLDDHGLELLEMMLVYD
AN4182.1  	IFR2ILGTPDETIWPGVTS---FPDFKPTFPKWKREDIQNVVPGLEEDGLDLLEALLEYD
          	***  **** *  **.**.   :****.:**** *:    :   *:: **:*** :* **

MG01362.1 	PAGRISAKQACNHPYFEDNTPAPPRR--------
NCU09778.1	PAGRISAKAACNHPYFEDYNPKPKSKYRDSSRYA
FG08468.1 	PAGRISAKGAFNHPYFEPHLAQKQASAQTNGYYH
AN4182.1  	PARRISAKQACMHPYFQHGSSYYSGRARRNGFH-
          	** ***** *  ****:   .        ..
```
